# Development of transplantable B-cell lymphomas in the MHC-defined miniature swine model

**DOI:** 10.1186/s12935-019-0954-3

**Published:** 2019-09-09

**Authors:** Alec R. Andrews, Zhaohui Wang, Robert A. Wilkinson, Jay A. Fishman, David H. Sachs, Nalu Navarro-Alvarez, Christene A. Huang

**Affiliations:** 1 Center for Transplantation Sciences, Department of Surgery, Massachusetts General Hospital, Boston, MA 02129 USA; 2 Department of Surgery, Division of Plastic & Reconstructive Surgery, Division of Transplant Surgery, University of Colorado School of Medicine, Aurora, CO 80045 USA; 3 Transplant Infectious Disease and Immunocompromised Host Program, MGH Transplant Center and Harvard Medical School, Boston, MA 02114 USA; 4 Columbia Center for Translational Immunology, Columbia University Medical Center, New York, NY USA; 5 Department of Gastroenterology, Instituto Nacional de Ciencias Médicas y Nutrición Salvador Zubirán, Mexico City, Mexico; 6 Department of Molecular Biology, Escuela de Medicina, Universidad Panamericana, Mexico City, Mexico; 7 University of Colorado Anschutz Medical Campus, Mail Stop 8621, Research Complex 2, 12700 E 19th Avenue, Aurora, CO 80045 USA

**Keywords:** B-cell lymphoma, preclinical large animal model, Serial transplantation

## Abstract

**Background:**

Establishment of transplantable tumors in clinically relevant large animals allows translational studies of novel cancer therapeutics.

**Methods:**

Here we describe the establishment, characterization, and serial transplantation of a naturally occurring B-cell lymphoma derived from a unique, highly inbred sub-line of Massachusetts General Hospital (MGH) major histocompatibility complex (MHC)-defined miniature swine.

**Results:**

The lymphoblastic cell line (LCL) originated from peripheral blood of a 2.5 year old female swine leukocyte antigen (SLA)^dd^-inbred miniature swine breeder demonstrating clinical signs of malignancy. Flow cytometric phenotypic analysis of subclones derived from the original cell line revealed surface markers commonly expressed in a B-cell lineage neoplasm. A subclone of the original LCL was transplanted into mildly-conditioned histocompatible miniature swine and immunocompromised NOD.Cg-*Prkdc*^*scid*^ *Il2rg*^*tm1Wjl*^/SzJ (NSG) mice. Tissue and blood samples harvested 2 weeks following subcutaneous and intravenous injection in a highly inbred SLA^dd^ pig were cultured for tumor growth and phenotypic analysis before serial transfer into NSG mice. Evidence of tumor growth in vivo was found in all tumor cell recipients. In vitro growth characteristics and surface phenotype were comparable between the original and serially transplanted tumor cell lines.

**Conclusions:**

These results indicate the feasibility of developing a large-animal transplantable tumor model using cells derived from spontaneously occurring hematologic malignancies within the highly inbred miniature swine herd.

## Background

Preclinical tumor studies have relied heavily on genetically modified or engineered small animals, which provide vital contributions in the identification of biological pathways involved in malignancy. Despite the data generated from small animals in oncology research, these models are faced with various limitations when translated to the clinic. At times, mouse studies have encountered difficulties predicting toxicity and pharmacokinetic interactions of candidate drug combinations [1, 2]. The mouse strains in which patient-derived tumors are studied in vivo may be significantly immunocompromised and often genetically modified to examine specific immune interactions in relation to tumor progression [2–4]. Large animals have greater genetic, physiological, anatomical, and immunological similarity to humans without some of the limitations of interspecies transfer of tumors.

The MGH MHC-defined miniature swine herd is a unique research animal resource for the study of transplantation and tolerance induction [5]. These miniature swine develop malignancies under conditions comparable to those underlying human disease, allowing for the cloning and characterization of tumor cell lineages endogenous to the herd [6–8]. The histocompatibility swine leukocyte antigen (SLA)^dd^ (herein referred to as DD) subline has now undergone 11 generations of brother-sister matings. Although the inbred DD subline is not yet fully syngeneic, animals from the 7th generation of this line were shown to be histocompatible by accepting heart and reciprocal skin grafts without immunosuppressive intervention [9]. Ideally, a tumor cell line derived from an inbred DD animal could be serially transferred within the herd, preserving native routes of oncogenesis. Here we describe the characterization of a spontaneous hematologic malignancy discovered in a naïve DD inbred miniature swine, as well as initial attempts at serial transplantation within this model.

## Materials and methods

### Miniature swine model

The MGH MHC-defined miniature swine model used in this study has been previously described [5]. A subline of the SLA^dd^ (DD) swine herd has undergone 11 generations of sequential brother-sister mating and have achieved a coefficient of inbreeding that exceeds 94%. This inbreeding has allowed for animals of the DD line to accept heart and reciprocal skin grafts without immunosuppression [9].

### Tissue and peripheral blood mononuclear cell processing

Heparinized whole blood samples were processed for peripheral blood mononuclear cell (PBMC) isolation by diluting blood with Hank’s balanced salt solution (HBSS, Thermo Fisher Scientific, Waltham, MA). Diluted blood was overlayed onto lymphocyte separation medium (Histopaque, Sigma-Aldrich, St Louis, MO) and underwent gradient centrifugation for cell isolation. The separated cell fraction was removed and washed twice with HBSS before resuspension in tissue culture medium. Tissue harvest from tumor recipients was performed in sterile manner. Cells were dissociated from connective tissue by mashing the piece of tissue with the flat end of a syringe plunger and passed through a 40 µm cell strainer placed over a 50 ml conical tube. Cells were washed twice with HBSS before being resuspended in tissue culture medium.

### Cell culture

Tumor cell lines of porcine origin were cultured in RPMI 1640 media with 12% fetal bovine serum (FBS), 2 mM Glutamine, 0.1 mM non-essential amino acid mixture, 1 mM sodium pyruvate, 10 mM HEPES buffer (4-(2-hydroxyethyl)-1-piperazineethanesulfonic acid), and 25 µM 2-Mercaptoethanol. All cultures were maintained at 37 °C with 5.0% CO_2_. Tumor culture viability was determined by stable expansion for at least 1 month, with passage occurring weekly. Subclones were established by limiting dilution. Each sublcone plate was examined for colonies every 10–14 days, and wells of interest were further expanded. Cultures were frozen in liquid N2 for storage at − 180 °C with a concentration of 1 × 10^7^cells/ml in cryoprotective media (Lonza, Walkersville, MD) diluted to a final concentration of 50% FBS.

### Growth curve

Cell turnover was characterized by establishing growth curves for each clone. Parent cultures were plated on 6-well plates in triplicate at an initial concentration of 5 × 10^3^ cells/well in 2 ml of culture media. Cell counts were performed daily using trypan blue exclusion dye (Corning, Manassas, VA) until a plateau was noticed. Culture media was replenished weekly.

### Flow cytometry

PBMCs, tissues, and cell cultures of both porcine and murine origin were analyzed by flow cytometry using either porcine specific or reactive antibodies directly conjugated to either Fluorescein or Biotin. Biotin labeled antibodies were detected using streptavidin phycoerythrin

(PESA; PharMingen, San Diego, California, USA). The list of antibodies tested include, CD1 (76-7-4 IgG2a), CD2 (MSA-4 IgG2a), CD3e (898H2-6-15 IgG2a), CD4 (74-12-4 IgG2b), CD5 (9G12 IgG1), CD8 (76-2-11 IgG2a), CD9 (1038H-4-6 IgM), CD11b (M1/70 IgG2b, BioLegend), CD16 (G7 IgG1), CD21 (B-ly4 IgG1), CD25 (231.3B2 IgG1), CD45RA (Fg-2F9 IgG1), CD172a (74-22-15 IgG1), CD80 (16-10A1 IgG, BioLegend), Class I D (2.12.3a IgM), Class II DR(40D IgG2b), Class II DQD (1052 h-5-21 IgG1), anti-mu heavy chain (5C9 IgG1), anti-lambda light chain (K139.3E1 IgG2a), CD79a (HM-57 IgG1, Bio-Rad). Cell labeling was performed as previously described [10]. Flow cytometry was tested with FACSCalibur (Becton–Dickinson, San Jose, CA). Data was analyzed using FlowJo software version 10.

### Histology and immunohistochemistry

Tissue samples were harvested from all animals at end of study necropsy and preserved in formalin for hematoxylin and eosin staining and immunohistochemistry. The list of antibodies used for immunohistochemistry include, CD45, CD79a (HM-57 IgG1, Bio-Rad), anti-lambda light chain (K139.3E1 IgG2a).

### Porcine lymphotrophic herpesvirus (PLHV) detection

PLHV-1 and PLHV-3 status was determined from stored PBMCs and tumor cells after recovery of DNA using the Gentra Puregene Kit (Qiagen Sciences, Germantown, MD), followed by Real Time TaqMan polymerase chain reaction (PCR) techniques described in detail [11, 12]. The PCR primers and probes used are listed in Table 1.

**Table 1:** PCR primers and probes for PLHV analysis

Reagents	PLHV-1	PLHV-3
Forward primer	5′ AAG GTG ACA TGC AAT GCT GTG 3′	5′ CTC AGG AAT TTG CAG CAC AT 3′
Reverse primer	5′ TGC AAT CTT GAG ACA GGG CA 3′	5′ ATG TCC AAT CGG TTC AGC AAT AC 3′
Probe	5′ FAM-TGG GTT CAC TGG TGT TGC ATC TGG TAT G-TAMRA 3′	5′ FAM-AGT TTT CTG TGA AGG TTT TAC TCA TCA CGG CC-TAMRA 3′

### In vivo transfer into miniature swine and NSG mice

**Fig. 1:**
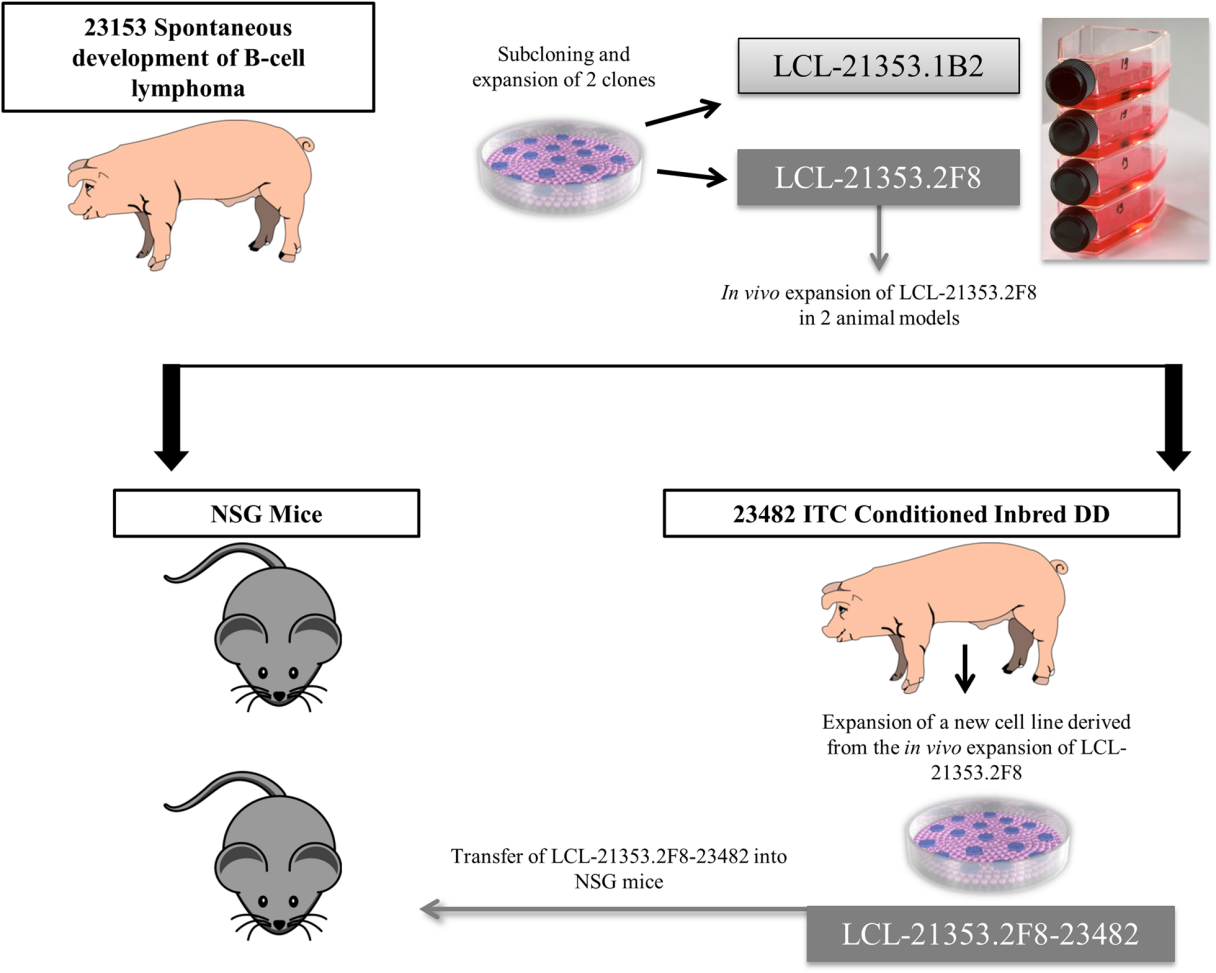
Schematic of experimental design. Flow chart including primary cell line harvest, scale up, and transfer within miniature swine and NSG mice

Figure 1 is a schematic representation of the experimental plan. In vitro tumor cultures were expanded over 1–2 months, pooled, and washed twice with phosphate buffered saline (PBS, Corning, Manassas, VA) before propagation in the pig model.

Porcine model: A mild conditioning regimen was initiated in the pig recipient 4 days prior to cell transplantation. The regimen included 4 days of intravenous (IV) anti-porcine CD3 immunotoxin [13] (0.05 mg/kg/dose administered twice daily), 100 cGy of total body irradiation, and 12 days of IV cyclosporine (target plasma range of 400-800 ng/ml). On day 0, the animal was injected with LCL-21353.2F8 at a dose of 1 × 10^8^ cells subcutaneously (SC) and 2.85 × 10^8^ cells/kg (4 × 10^9^ total cell dose) IV through an indwelling catheter placed in the external jugular vein.

Mouse model: Tumor cultures were removed from storage, propagated through two passages, and washed twice with PBS before transfer into NOD.Cg-*Prkdc*^*scid*^ *Il2rg*^*tm1Wjl*^/SzJ (NSG) mice (Jackson Laboratory, Bar Harbor, ME). Mice were given IV and or intraperitoneal (IP) injections as follows: Group 1 (n = 6) received 1 × 10^7^–2 × 10^7^ IP and 1 × 10^7^ cells IV from LCL-21353.2F8 cell line. Group 2 (n = 4) received either no or 0.5 × 10^7^ cells IP and 1 × 10^7^ cells IV from LCL-21353.2F8-23482 cell line.

All animal experiments were approved by and in compliance with Massachusetts General Hospital (MGH) Institutional Animal Care and Use Committee (IACUC). MGH is an Association for Assessment and Accreditation of Laboratory Animal Care (AAALAC) recognized research institution.

## Results

### Characterization of spontaneous tumor cell lines

**Fig. 2:**
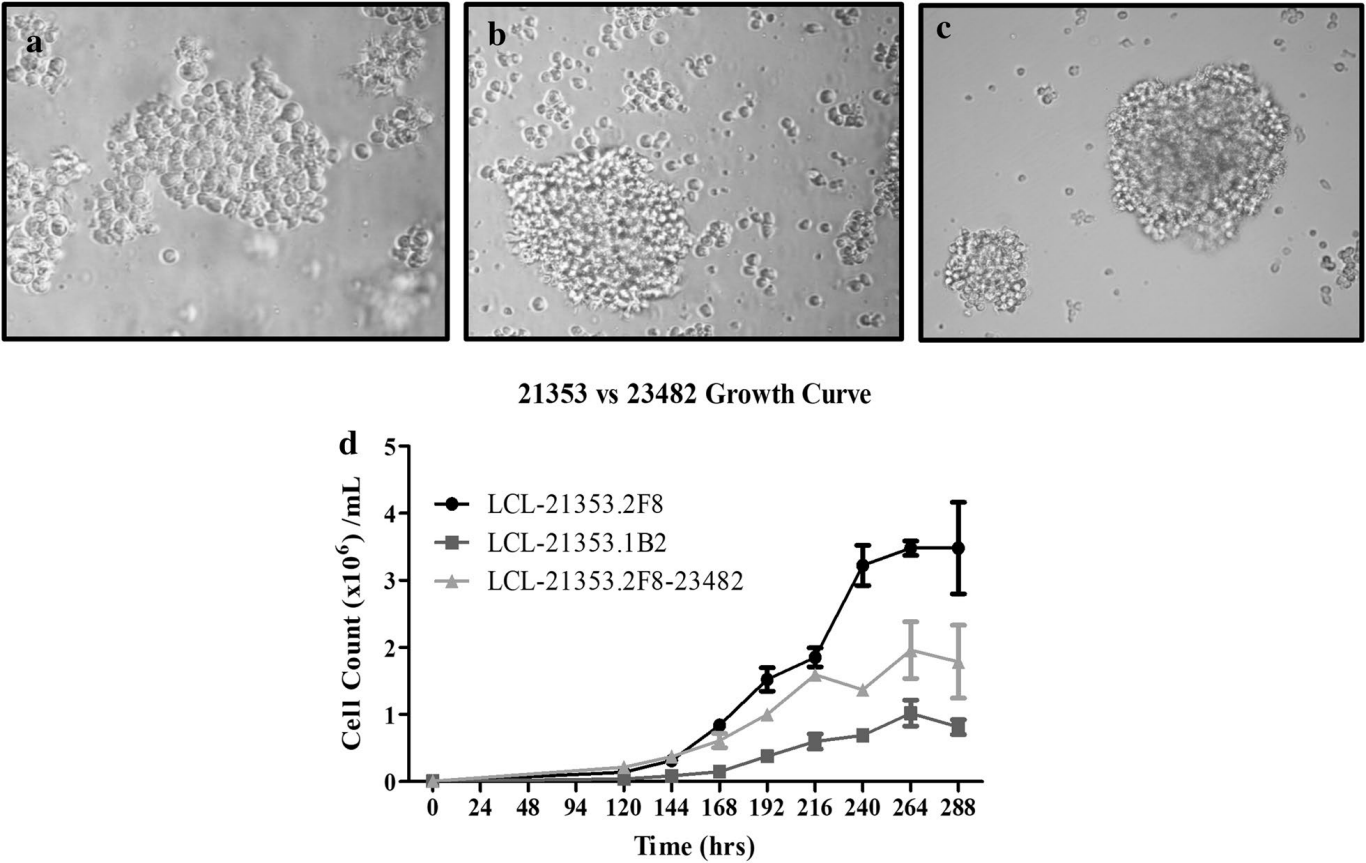
Growth characteristics of the swine derived tumor cell lines. **A** LCL-21353.1B2 culture image taken after third passage. **B** LCL-21353.2F8 culture image taken after third passage. **C** 21353.2F8-23482 culture image taken after second passage. **D** Growth curve comparing the three cell lines. All microscopy was performed using Nikon Eclipse TE2000-U microscope under 20× magnification at room temperature. Images processed using Adobe Photoshop 6.0 (Adobe Systems)

**Fig. 3:**
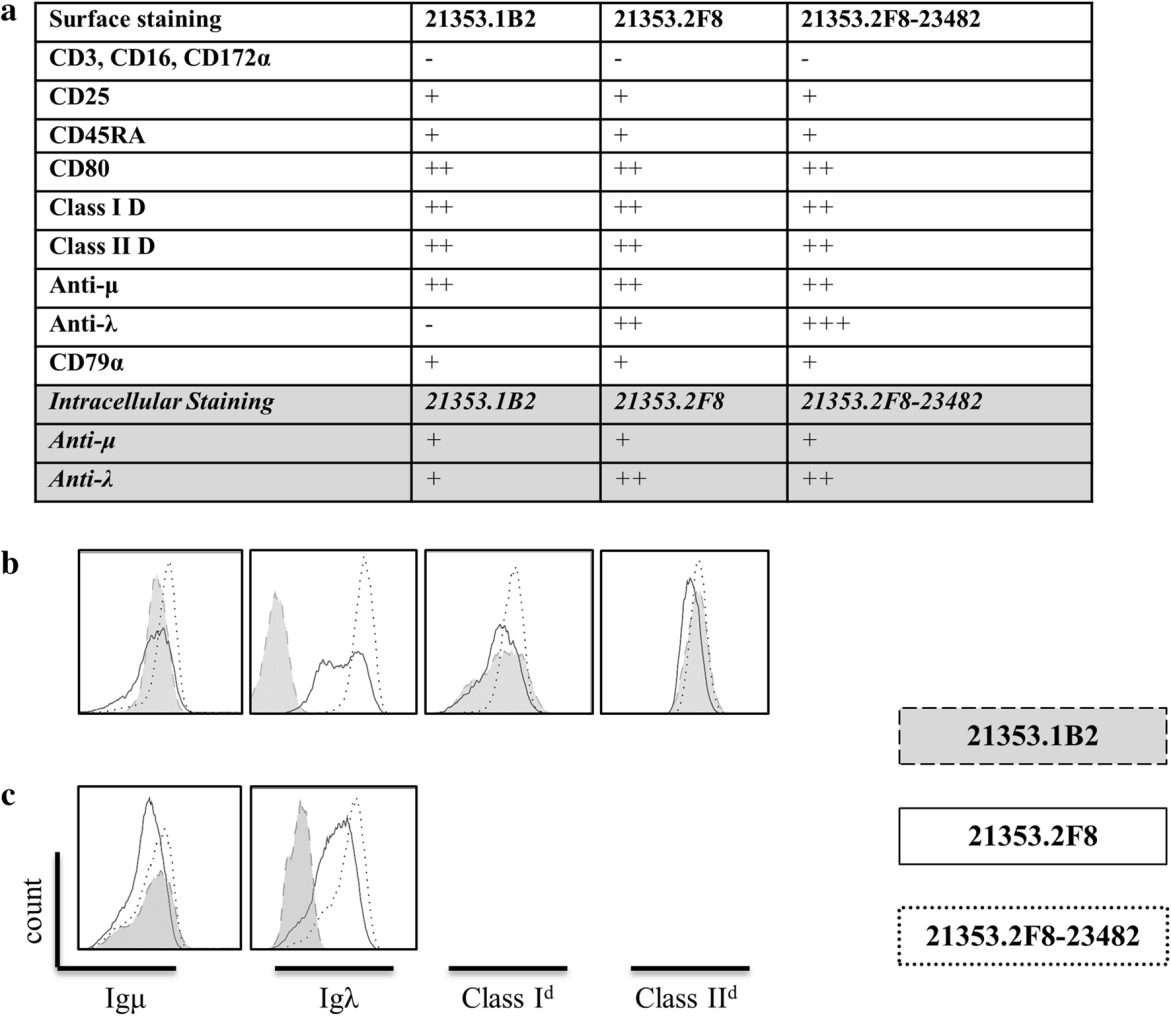
Flowcytometric analysis of tumor cell lines. **a** Descriptive list of staining characteristics for the three swine derived tumor cell lines. No change from negative control (−), shift between 10^1^ and 10^2^ (+), shift between 10^2^ and 10^3^ (++), shift greater than 10^3^ (+++). **b** Histograms depicting surface staining of the three tumor cell lines for characteristic markers of a B-cell lineage neoplasm. **c** Histograms depicting intracellular staining of mu heavy and lambda light chain. Gray filled histograms with dashed outline represent LCL-21353.1B2, white filled histograms with solid outline represent LCL-21353.2F8, and white filled histograms with dotted outline represent LCL-21353.2F8-23482. Non-viable cells were removed using 7-AAD, gate placed on lymphocytes population identified in the forward/side-scatter prior to analysis

Malignancy was suspected in a 2.5 year old female generation (G)11 inbred DD animal 21353, which presented with weight loss, mild head tilt, lethargy, and an elevated white blood cell count. Peripheral blood samples were drawn, processed, placed in culture and subcloned by limiting dilution in attempt to establish a tumor cell line in vitro. Subclone LCL-21353.2F8 was successfully propagated and selected for further study. Examination of cultures by light microscopy revealed large clusters of cells aggregated in suspension with rapid doubling time and stable growth characteristics, depicted in Fig. 2A–C. Immunophenotyping by flow cytometry identified MHC class I expression as being comparable to normal whole blood lymphocyte population, while all cells stained positive for MHC class II, representing a homogenous population (Fig. 3a, b). Lack of CD16 and CD172a surface markers typically expressed in myeloid lineage, as well as high expression of mu heavy chain and lambda light chain, suggests the clone is derived from a B-cell malignancy (Fig. 3a, b). Elevated expression of lymphocyte activation markers CD80 and CD45RA were also observed. Another subclone derived from animal 21353, LCL-21353.1B2, demonstrated less robust growth characteristics (Fig. 2D), as well as the absence of lambda light chain surface expression (Fig. 3a, b). Intracellular staining revealed LCL-21353.1B2 was positive for cytoplasmic lambda light chain (Fig. 3c).

Porcine lymphotrophic herpesvirus (PLHV) status was examined for 21353 PBMC banked shortly after disease onset, and was found to be positive for both PLHV-1 and PLHV-3 (primers and probes in Table 1). Following subcloning and long-term culture, subclones LCL-21353.2F8/1B2 derived from 21353 PBMC remained PCR positive for PLHV-3 but were negative for PLHV-1 (Additional file 1: Figure S1).

### Transplantation into immunosuppressed MHC-matched miniature swine

After a subcloned and characterized tumor line was established, we sought to propagate growth in vivo using a histocompatible miniature swine, G10 inbred DD animal 23482 (see Additional file 2: Table S1 for general characteristics of the animals). The recipient received mild conditioning prior to IV and SC transplantation of cell line LCL-21353.2F8 (see methods for detailed conditioning regimen). Cyclosporine levels remained within therapeutic range throughout the protocol. Unfortunately, the animal succumbed to acute respiratory complications 11 days after receiving tumor cell injections. The SC injection site was biopsied during necropsy, and a solid mass was removed.

The SC mass was harvested and cultured; after multiple passages, the remaining cells proliferated with similar physical and phenotypic characteristics to those of the primary cell line LCL-23153.2F8 (Fig. 2C, D). Activation markers CD80 and CD45RA remained consistent (Fig. 3a), while mu heavy chain intensity decreased and anti-lambda light chain increased after transplantation (Fig. 3a–c). Of note, LCL-23153.2F8-23482 displayed more homogeneity in both MHC class I and lambda light chain surface expression (Fig. 3b). In addition to the cell line derived from the SC site, tumor cultures were established from various sources including the spleen and peripheral blood, which were harvested at the time of necropsy. (Data not shown)

**Fig. 4:**
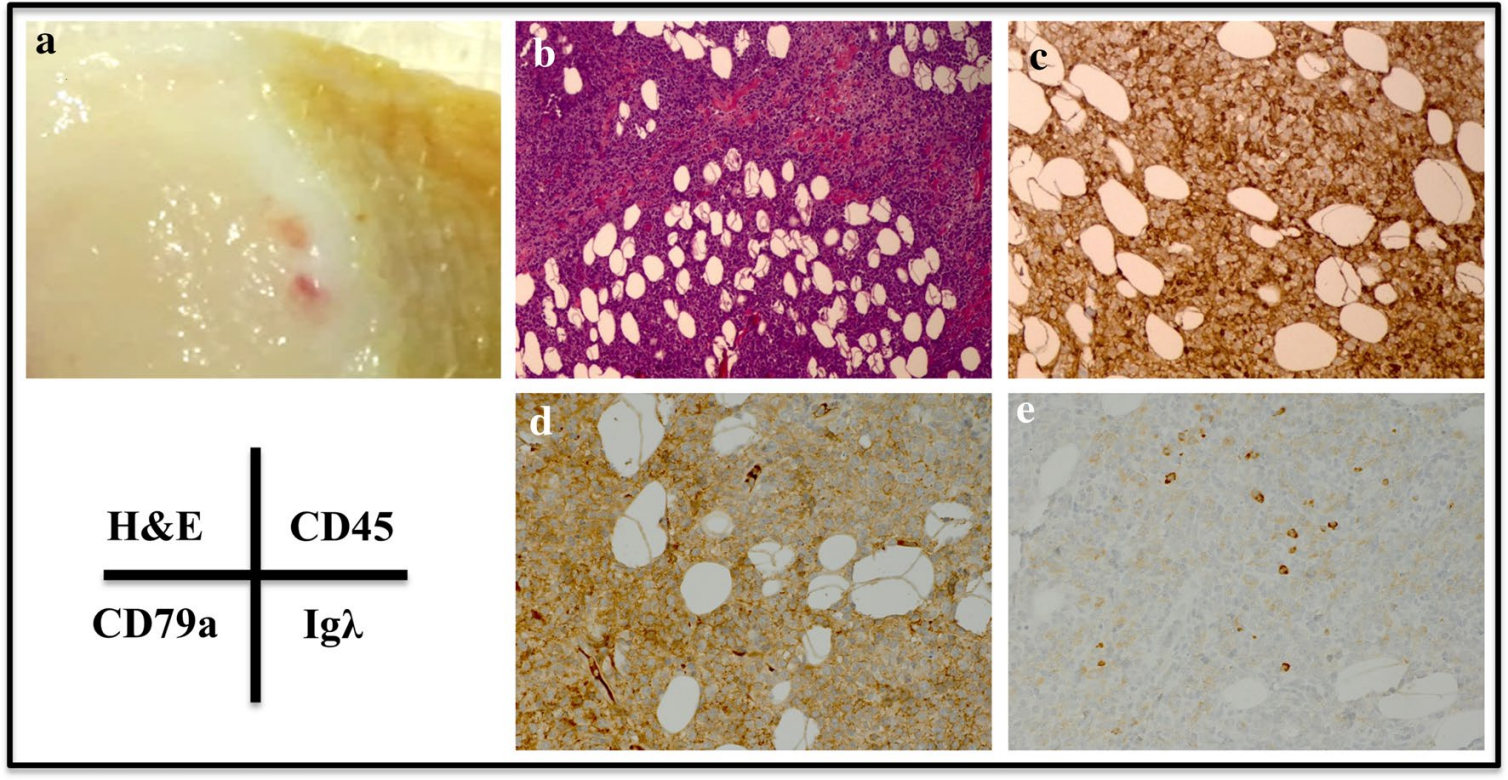
Histologic analysis of tumor injected swine 23482. **A** Gross image of subcutaneous tumor injection site harvested at necropsy (11 days post-injection). **B** H&E staining, **C** CD45 staining, **D** CD79a staining, **E** lambda light chain staining of punch biopsy taken from the subcutaneous site 7 days after injection. All images were taken at 20× magnification

Tumor formation can be visualized macroscopically in the mass removed from the SC injection site (Fig. 4A). When analyzed histologically, tumor infiltrates became apparent in this space (Fig. 4B–E). Figure 4C, clearly shows an abundance of hematopoietic cells in this region, as characterized by CD45 staining, while CD79a cytoplasmic staining was evident throughout (Fig. 4D). Lambda light chain immunostaining was less convincing, as only a limited number of cells within the sample were positive (Fig. 4E).

### Transfer into NSG mice

**Fig. 5:**
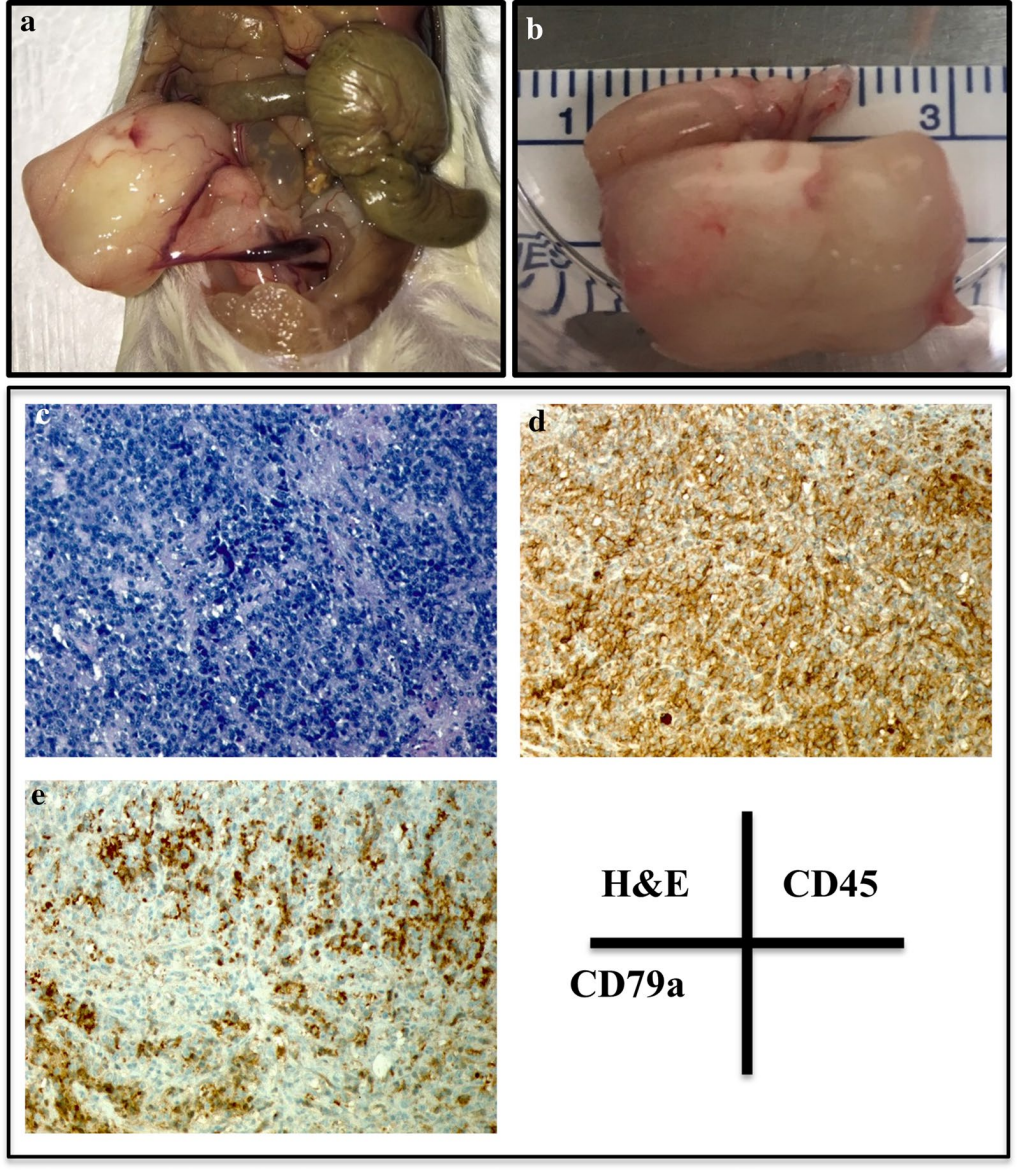
Histologic analysis of Group 1 mice (recipients of LCL-21353.2F8). **A** Image of intact vasculature found on the abdominal tumor mass from mouse 2588. **B** Measurement of the isolated mass from mouse 2588. **C** H&E, **D** CD45, and **E** CD79a immunostaining performed on an abdominal tumor mass from mouse 2589 using porcine specific antibodies. All images were taken at 20× magnification. Staining was performed on masses harvested 70 days after tumor injections

**Fig. 6:**
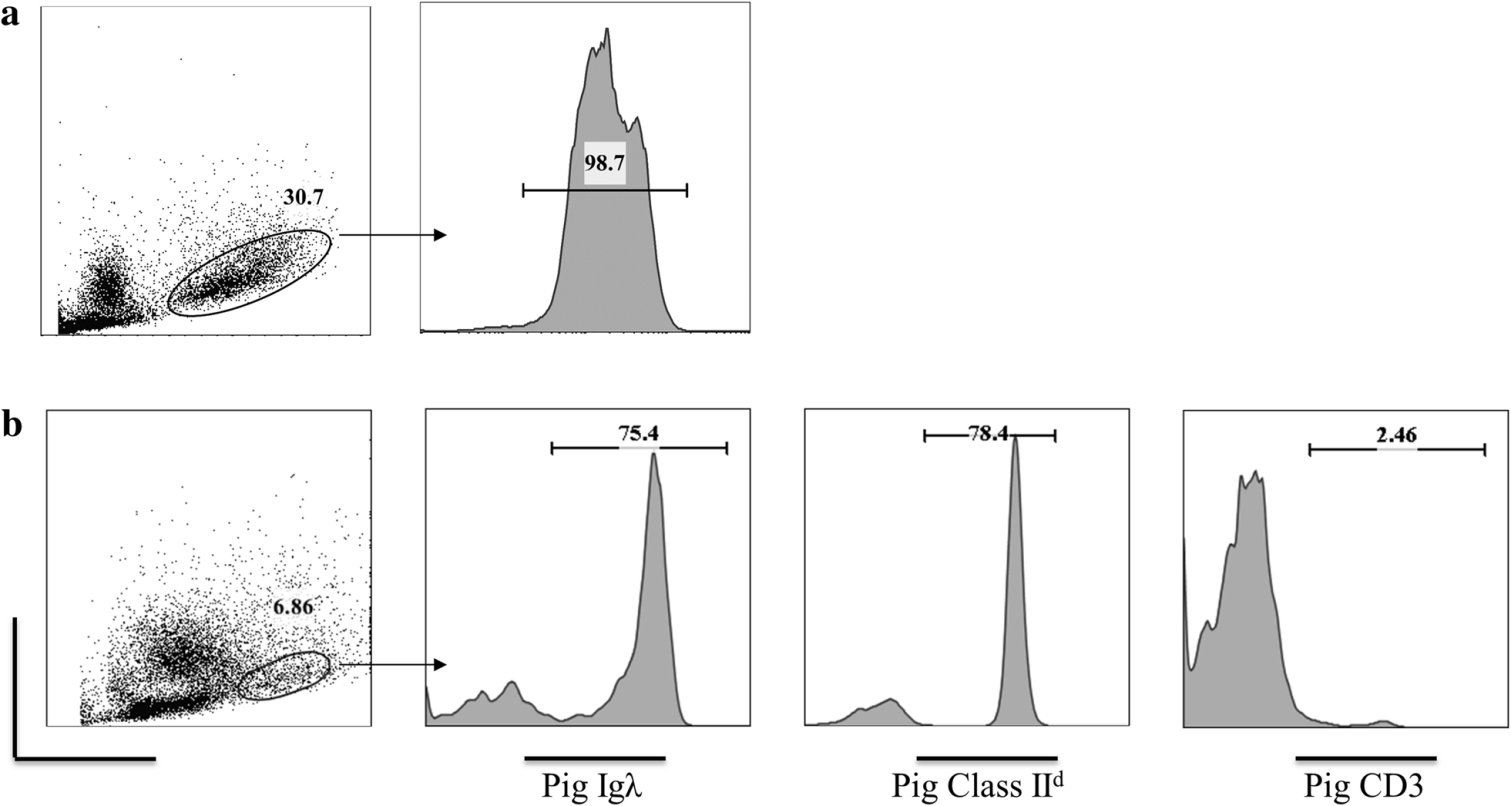
Flow cytometric evidence of LCL-21353.2F8-23482 in NSG spleen. **a** Representative phenotyping of a small abdominal tumor mass harvested from mouse 2560 which received LCL-21353.2F8 tumor injections. **b** Presence of tumor cells found in the spleen of mouse 44, despite the lack of solid mass formation in all Group 2 mice. Non-viable cells were removed using 7-AAD, gate placed on large lymphocytes in the forward/side-scatter shown above

To confirm the in vivo viability of each swine derived cell line, NSG mice were selected to receive both primary and secondary swine tumor cells at varying doses and routes of administration (described in Tables 2 and 3). Recipients of LCL-21353.2F8 manifested clinical signs of malignancy within 1 month of injection. The symptoms ranged from mild hind limb paralysis to hunched posture and decreased activity. Solid masses were found throughout the abdomen of each recipient upon necropsy (Fig. 5A, B). Mass size corresponded with the end of study date, at which time ~ 2 cm inguinal tumors were identified in all animals that continued until day 70 (Fig. 5A, B). In each of these cases, the solid tumors were vascularized and developed in strikingly similar fashion, surrounding adjacent organs without the formation of ascites. Tumor cells were demarcated within the mass by histologic analysis (Fig. 5C–E). The cells were large in size, with scant cytoplasm and the characteristics of a lymphocytic cell (Fig. 5C). The tumor cells were positive for CD45 confirming their hematopoietic origin (Fig. 5D) and positive for CD79a (Fig. 5E), validating their derivation from the original tumor cell line, which demonstrated CD79a staining in vitro. Recipients with earlier end of study dates had less significant gross tumor formation, with < 1 cm masses observed sporadically throughout the abdomen. The abdominal mass of an early terminated mouse was examined by flow cytometry using porcine specific anti-lambda light chain. A considerable shift was noted in the tumor mass, which stained brightly positive (Fig. 6a).

**Table 2:** Recipients of LCL-21353.2F8 (group 1 mice)

Mouse	IV dose	IP dose	Clinical assessment	Day
2587	1 × 10^7^	2 × 10^7^	Mild hind limb paralysis, decreased activity	70
2588	1 × 10^7^	1 × 10^7^	Mild hind limb paralysis, decreased activity	70
2589	1 × 10^7^	1.8 × 10^7^	Mild hind limb paralysis, decreased activity	70
2590	1 × 10^7^	1 × 10^7^	Hunched posture, decreased activity	55
2577	1 × 10^7^	1 × 10^7^	Hunched posture, decreased activity	55
2560	1 × 10^7^	1 × 10^7^	Mild Hind limb paralysis, hunched posture, decreased activity	41

**Table 3:** Recipients of LCL-21353.2F8-23482 (group 2 mice)

Mouse	IV dose	IP dose	Clinical assessment	Day
41	1 × 10^7^	0.5 × 10^7^	Moderate hind limb paralysis, hunched posture, decreased activity	35
42	1 × 10^7^	0.5 × 10^7^	Moderate hind limb paralysis, hunched posture, decreased activity	35
43	1 × 10^7^	–	Moderate hind limb paralysis, hunched posture, decreased activity	35
44	1 × 10^7^	0.5 × 10^7^	Hunched posture, decreased activity	62

Unlike the primary tumor line, secondary transfer of LCL-21353.2F8-23482 into NSG mice did not lead to solid mass formation by end of study. Despite the absence of solid tumor masses in this cohort, 3 of 4 mice that received LCL-21353.2F8-23482 developed moderate hind limb paralysis within 2 weeks of cell injection. The presence of porcine tumor cells was confirmed at time of sacrifice by flow cytometry of isolated splenocytes. Figure 6b demonstrates a brightly stained population of surface porcine Ig lambda light chain and porcine MHC Class II among splenocytes from an NSG recipient of LCL-21353.2F8-23482.

## Discussion

Tumor lines of natural origin harvested from large animals, recapitulate disease onset and progression seen clincially. This lab has previously described the development of lymphoma in immunosuppressed, inbred miniature swine in association with PLHV-1 and PLHV-3 infections [11, 14–16]. In this report, we describe both the IV and SC transplantation of a naturally occurring porcine B-cell lymphoma, demonstrating the practicality of serial tumor transfer within the inbred DD swine herd, when coupled with a mildly immunosuppressive conditioning protocol previously described for the induction of solid organ transplantation tolerance and establishment of mixed chimerism in the MHC-defined miniature swine [17, 18]. The spontaneous B-cell lymphoma originated in a G11 inbred DD swine and was transferred to a histocompatible (DD) G10 relative. It is important to note that even in inbred mice, most spontaneous tumors are not directly transplantable to syngeneic immunocompetent mice [19, 20]. To facilitate tumor cell establishment in vivo, we subjected the recipient to mild conditioning before tumor cell transfer. This minimally toxic conditioning regimen allowed the survival of tumor cells in peripheral blood, spleen, and early onset SC mass formation. The injected pig was sacrificed 11 days after cell transplant due to acute respiratory distress presumably related to pulmonary embolus resulting from the indwelling catheter. Due to lack of additional and appropriately sized DD swine at the time, we were unfortunately unable to transfer these cells into an additional swine recipient.

The distinguishable feature between all three cell lines examined in this study is lambda light chain surface expression. Clone LCL-21353.1B2 displayed an interesting phenotype of mu heavy chain surface expression with a notable lack of surface lambda light chain, differentiating this cell line from the other two. However, intracellular staining revealed that the population was positive for cytoplasmic lambda light chain. Lack of surface membrane immunoglobulin light chain is well documented in human B-cell lymphomas, although the etiology of this phenomenon remains undefined [21, 22]. It is presumed that errors can occur in various points along the spectrum of B-cell development, resulting in a malignant phenotype and expansion of a B-cell clone. This expansion can be evaluated clinically by flow cytometry, focusing on perturbations in kappa and lambda surface expression ratio within the total B-cell population [21–24]. Since LCL-21353.1B2/2F8 only differ in their surface lambda light chain expression, it is plausible that LCL-21353.1B2 represents a cell subset that underwent further mutation rendering the cells incapable of transporting the light chain to the membrane surface. This mutation could have effected post-translational modifications or biochemical interactions with the immunoglobulin heavy chain at the cell surface. Further molecular analysis will need to be performed in order to clarify this point. In addition to the lack of surface Igλ expression, LCL-21353.1B2 has noticeable growth deficits compared to LCL-21353.2F8, but maintains long term survival in vitro.

Samples from the two cell lines expressing surface lambda light chain (LCL-21353.2F8 and LCL-21353.2F8-23482) were transferred into NSG mice leading to debilitating paralysis in both mice cohorts. Group 1 recipients developed mild hind limb paralysis in all but two cases, while Group 2 recipients advanced to moderate hind limb paralysis in all but one case. When comparing the two injections it is noted that both groups received identical IV cell doses, in contrast to IP doses, which varied. Therefore, it appears paralysis onset and severity was increased after serial transfer through SC injection in swine, and is most likely associated with IV injection in the mice. A previous study examined the transplantation of human B-cell lymphoma lines into NSG mice, leading to similar paralytic outcomes in which hind limb paralysis was thought to be caused by IV exposure to tumor cells, which subsequently localized to the spinal cord [25]. Clinically, B-cell lymphomas presenting with neurological symptoms are correlated with poor prognosis, as they are most commonly identified as progressive or late stage disease [26, 27]. Applying this definition to our mouse model indicates that LCL-21352.2F8-23482 could be interpreted as more aggressive after transfer in the pig model, due to early onset of paralysis in 75% of NSG recipients.

Although paralysis was more severe in LCL-21353.2F8-23482 recipients, solid tumor formation did not occur in these mice. Tumor masses were only observed in NSG mice that received the parent tumor cell line LCL-21353.2F8. This finding could be due to the decreased IP cell doses given to Group 2 mice, or may be caused by the increased surface expression of both MHC class I and II observed in LCL-21353.2F8-23482 (Fig. 3b), or other undetectable modifications affecting the ability to form solid tumors.

Previous translational drug trials have utilized SC tumor growth as a readout for efficacy, where drug candidates are injected locally and function is interpreted based on the reduction in mass size [2, 28]. These preclinical studies are generally carried out in mice, which provide the benefit of reduced study drug cost, the ability to view SC growth with the unaided eye, and ease of repeated experiments due to available animal numbers. In the pig model, the total number of injection sites can be increased within the same experimental animal, allow for repeated infiltrate analysis through non-invasive punch biopsies, and scale drug dosing to physiologically relevant parameters used in humans. Porcine models historically generate reliable preclinical toxicity data due to comparable size and anatomy between humans and pigs, as well as similarities found in cytochrome P450 protein function [29]. Similar studies in other large animal models (dogs) have been limited due to lack of inbred histocompatible animals, and the requirement for intensive immunosuppression [30, 31]. Our swine B-cell lymphoma model therefore has the capacity to merge these two areas of research into a highly translational model.

Recent progress has been made in porcine tumor studies through human xenograft into immunodeficient pigs [32], and the virally induced Oncopig model. The Oncopig incorporates heterozygous mutations in common tumor associated genes *KRAS*^*G12D*^ and *TP53*^*R167H*^ [33, 34]. Benefits of this model are clear, since MHC haplotypes can be controlled reducing the need for immunosuppression, and malignancy can theoretically be triggered in any cell type of choice through the transfection of a Cre-recombinase in vitro prior to transplant [33, 34]. By harnessing germline mutations, oncogenesis is directed by the activation of the oncogene KRAS and a defective copy of the p53 tumor suppressor gene, thus reflecting consistent features of the human cancers they model [35]. Our tumor study adds to this field through the transfer of an unedited hematologic neoplasm that retains phenotypic markers found in the primary cell line even after transplantation within the histocompatible inbred DD swine herd; an alternative to genetically engineered or xenogeneic cell lines used in the same species.

## Conclusions

We have described a spontaneous hematologic malignancy in the MHC-defined miniature swine, and demonstrated the viability of the resulting tumors by in vitro culturing as well as by in vivo passage in immunocompromised mice and immunosuppressed swine. These studies represent a promising advance in the development of a large animal preclinical tumor model.

## Supplementary information

**Additional file 1: Figure S1.** PLHV status. PLVH-1 and PLVH-3 status time line in PBMC harvested from animal 21353 and in the 2 subclones (LCL-21353.1B2 and LCL-21353.2F8) obtained from it.

**Additional file 2: Table S1.** General Characteristics of porcine used.

## Data Availability

The authors declare that all relevant data supporting the findings of this study are available within the article information files.

## Abbreviations

LCL: lymophoblastic cell line
SLA: swine leukocyte antigen
PLHV: porcine lymphotropic herpesvirus
MHC: major histocompatibility complex
PLHV-1: porcine lymphotropic herpesvirus 1
PBMC: peripheral blood mononuclear cells
HBSS: Hank’s balanced salt solution
PCR: polymerase chain reaction
SC: subcutaneous
IV: intravenous
