# Supplementary figures and images for: Development of transplantable B-cell lymphomas in the MHC-defined miniature swine model
Source: Cancer Cell Int. 2019 Sep 9;19:236. doi: 10.1186/s12935-019-0954-3 (PMC6734256; doi:10.1186/s12935-019-0954-3)

## Slide 1
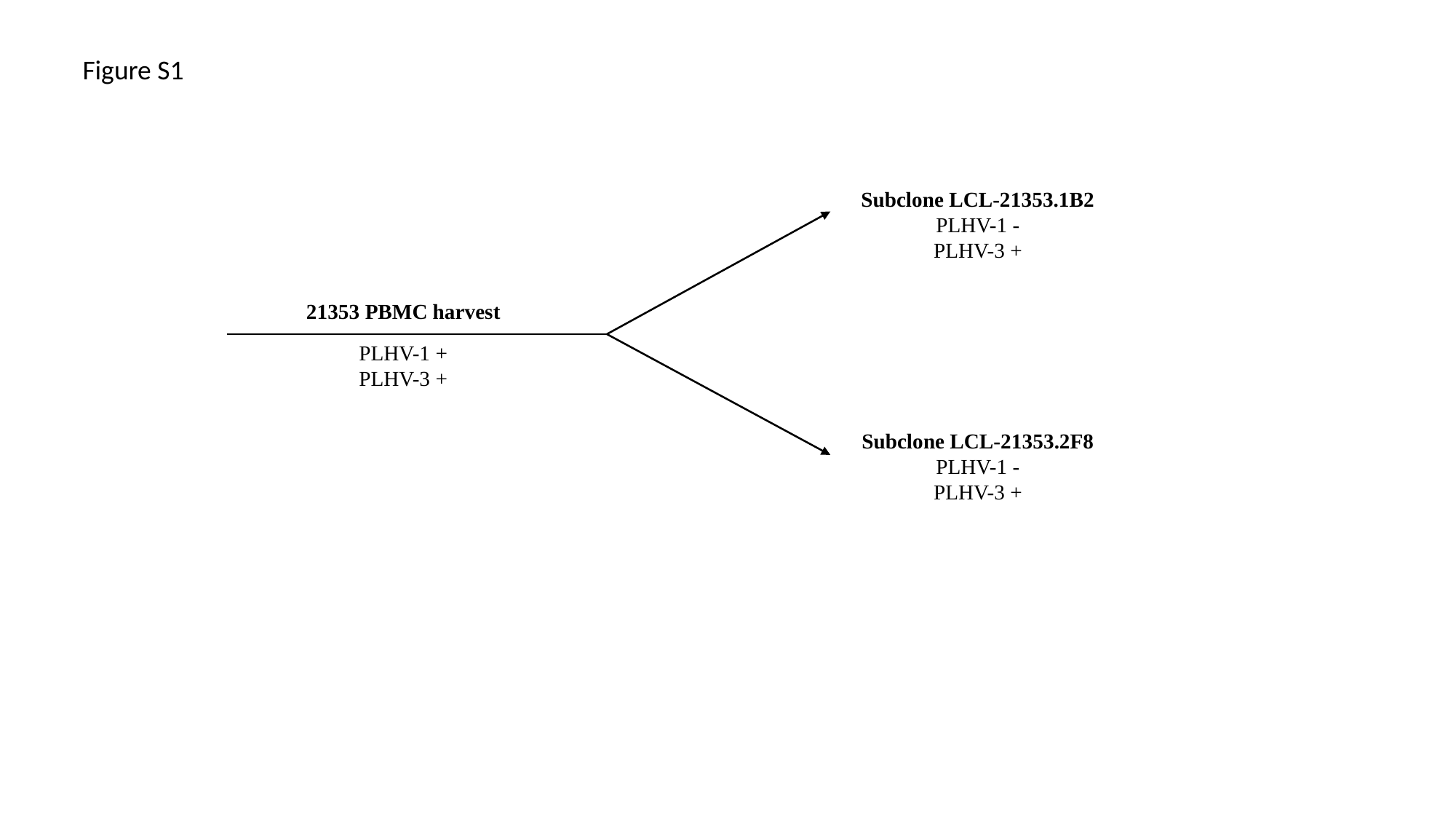

Figure S1
Subclone LCL-21353.1B2
PLHV-1 -
PLHV-3 +
21353 PBMC harvest
PLHV-1 +
PLHV-3 +
Subclone LCL-21353.2F8
PLHV-1 -
PLHV-3 +

Supplement: Supplementary file 1 — Additional file 1: Figure S1. PLHV status. PLVH-1 and PLVH-3 status time line in PBMC harvested from animal 21353 and in the 2 subclones (LCL-21353.1B2 and LCL-21353.2F8) obtained from it. [file 12935_2019_954_MOESM1_ESM.pptx]
