# Supplementary material for: Development of transplantable B-cell lymphomas in the MHC-defined miniature swine model
Source: Cancer Cell Int. 2019 Sep 9;19:236. doi: 10.1186/s12935-019-0954-3 (PMC6734256; doi:10.1186/s12935-019-0954-3)
Supplement: Supplementary file 2 — Additional file 2: Table S1. General Characteristics of porcine used. [file 12935_2019_954_MOESM2_ESM.docx]

Table S1. General Characteristics of porcine used

| **Animal** | **Age** | **Sex** | **PLHV-1 status** | **PLHV-3** | **Mass tumor localization** |
| --- | --- | --- | --- | --- | --- |
| **21353** | 2.5y | F | + | + | PBMC |
| **23482** | 4mo | F | Not Assessed | Not Assessed | SC tissue, PBMC, Spleen |
